# High-Origin Radial Artery: Frequency and Morphology in Midwestern Cadaveric Specimens

**DOI:** 10.7759/cureus.99285

**Published:** 2025-12-15

**Authors:** Umair Naseem, Michael Pollack, Khalid Khan, Peyton Grant, Autumn Coon, Sunny Patel, Shanu Markand

**Affiliations:** 1 Anatomy, A.T. Still University, Kirksville, USA

**Keywords:** anatomical variation, bifurcation, brachial artery, radial artery, upper limb

## Abstract

Purpose: The radial artery (RA) and ulnar artery are continuations of the brachial artery (BA) at the level of the antecubital fossa and supply most structures in the forearm and hand. Given variations in vessel nomenclature at anatomical landmarks, this study aimed to determine the frequency, level, and morphological characteristics of a high-origin RA in Midwestern cadaveric specimens.

Methods: To identify these variations, we evaluated the branching of the BA in 31 US Midwestern donor bodies from A.T. Still University’s Kirksville College of Osteopathic Medicine Gift of Body Program. When abnormal BAs were identified, we characterized the variation by describing the RA. Specifically, we identified the RA by tracing it to the distal lateral ventral arm, recorded its length in centimeters, and photographed its origin and course.

Results: Sixty-one upper limbs were evaluated for BA variations. Of these, seven (11.5%) had variations in the bifurcation point, and all originated higher than the antecubital fossa from the axillary artery. Four (57.1%) variations were found in the right limb and 3 (42.9%) in the left.

Conclusion: These results suggest that just over a tenth of BA exhibit variations in arterial branching patterns and emphasize the importance of understanding anatomical variations, particularly in relation to procedures such as coronary artery angiography and percutaneous arterial interventions.

## Introduction

The radial artery (RA) is the lateral continuation of the brachial artery (BA) in the upper limb, typically bifurcating into the radial and ulnar arteries about 1 cm distal to the flexor crease of the antecubital fossa [1]. The BA, in turn, is the distal continuation of the axillary artery and changes its name at the lower border of the teres major [2]. Given the intricate nature of the upper limb vasculature, embryogenesis plays a crucial and highly regulated role in determining the origin and branching of these arteries [3]. The superficial BA forms an anastomosis with a trunk that gives rise to the RA’s deep origin in the primitive axial artery [4]. Over time, the definitive arterial pattern of the upper limb develops from the primitive capillary plexus, with the dominant vascular channels differentiating [5].

Distally, the ulnar artery courses anteromedially through the forearm and passes through Guyon’s canal, supplying blood to most of the wrist flexors, the extensor carpi ulnaris, and the muscles of the hypothenar compartment [6]. In contrast, the RA follows an anterolateral course deep to the brachioradialis muscle before curving posterolaterally at the wrist and traversing the anatomical snuffbox; this anatomical structure is important for such procedures as creating arteriovenous fistulas between the RA and cephalic vein [1]. The RA is also crucial for supplying blood to the carpal bones, thumb, and the lateral aspect of the index finger [7]. 

Because of its accessibility and postoperative hemostasis, the RA is a commonly used blood vessel for various surgical and cardiovascular procedures [8]. It is also widely used for pulse monitoring since it is relatively superficial and easy to palpate [9]. Similarly, variations in the branching of the BA are important for medical professionals performing procedures such as retrograde catheterization or harvesting for bypass grafts [10]. When these professionals are aware of possible variations, they can minimize complications and improve accuracy during the procedure. In addition, for patients with BA variations, some procedures, including transradial access, are contraindicated and require a transfemoral approach [8]. 

In general, anatomical variations in the RA arise from the embryological development of the upper limb vasculature, resulting from differential enlargement and regression of arterial segments [8]. The superficial BA plays a consistently important role in the development of the typical vasculature of the upper limb [11]. Typically, variations in the origin of the vasculature of the upper limb arise from the dominant vascular channels. As a result, the RA may have a high or low origin. In addition, many other genetic regulators contribute to deviations from the typical origin of the BA and its branching [5]. Studies have shown that bifurcation variations in the BA are common [11,12]. One of the most frequently observed variations is in the brachioradial artery, where the RA originates from either the brachial or axillary artery [12]. Previous research involving donor bodies has reported that this variation ranges from 9.2% to 13.21% [11,12]. However, most of these studies have been conducted on the general population, and there is limited data specifically about individuals from the Midwestern United States [11,12]. Given changes in vessel nomenclature at various anatomical landmarks and the limited data on RA origin in this population, the purpose of the current study was to determine the frequency, anatomical level, and morphological characteristics of a high-origin RA.

## Materials and methods

To identify these potential variations, we evaluated the branching of the BA in 31 US Midwestern donor bodies from A.T. Still University’s Kirksville College of Osteopathic Medicine Gift of Body Program. First-year osteopathic medical students dissected donor bodies, but the study authors performed additional dissections for better visualization. Donor bodies with missing upper limbs and those that had the RAs or BAs severed for dissection or practical tagging were excluded from the study. Of the 31 donor bodies, 61 BAs were evaluated; one RA was excluded because of multiple abrasions in the proximal upper limb that abruptly terminated the RA. All procedures in the current study were considered exempt by the A.T. Still University-Kirksville Institutional Review Board (IRB #SM20240806-001). 

**Figure 1:**
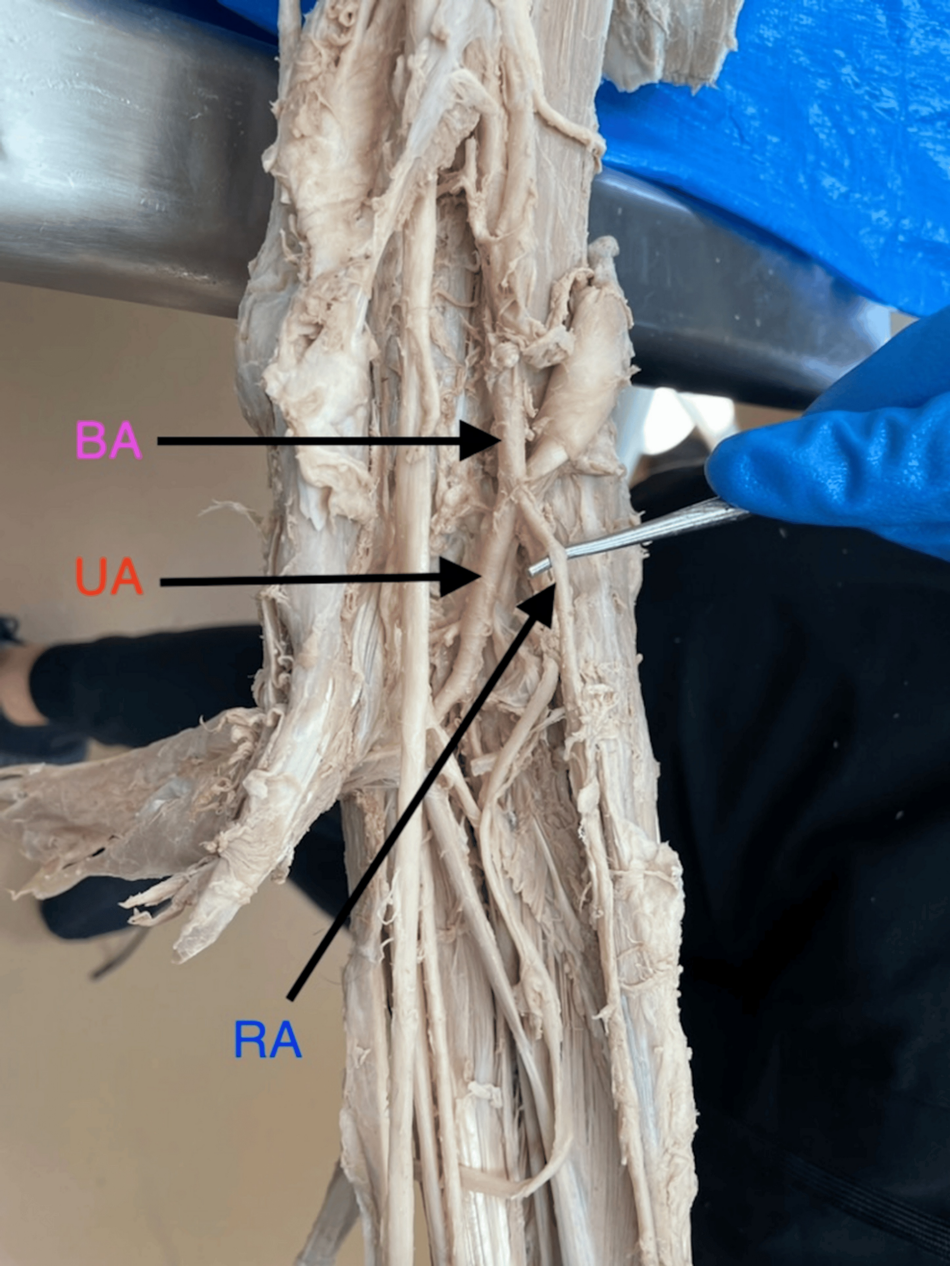
Typical branching pattern of the brachial artery. The brachial artery bifurcates into the radial artery and ulnar artery. BA, brachial artery; RA, radial artery; UA, ulnar artery.

The study began by identifying the BA's location. When an aberrant branching pattern was observed, we characterized the variation by describing the RA. Specifically, we identified the RA by tracing it to the distal lateral ventral arm. It was distinguished from the ulnar artery by its lateral course and ventrolateral appearance in the distal antebrachium (Figure 1). Next, the length of the RA was recorded in centimeters using a measuring tape. For this study, RA length was defined as the linear distance from the BA bifurcation to the designated cubital fossa reference point (the midpoint between the medial and lateral epicondyles). The origin and course of the RA for each abnormal bifurcation were then photographed using an Apple iPhone 12 Pro. To aid identification in the photographs, a blue pipe cleaner was tied around the RA, and a red one was tied around the ulnar artery. We also recorded details about the origin and course. 

To summarize the observed variations in the BA, we recorded the sex of the donor body, the side of the aberrant branching pattern, and the donor’s age at death. Frequency and percentage were used to determine the proportion of variations in our study sample, and the mean and standard deviation (SD) were used to summarize the length of the RA from the origin of the bifurcation to the antecubital fossa. 

## Results

Six of the 31 donor bodies had at least one aberrant branching pattern of the BA in a limb, and one of these had a bilateral branching pattern. Four (66.7%) of the donor bodies were males, and two (33.3%) were females (Table 1). Among the seven observed BA variations (N = 7; 11.5%) identified in our sample of 61 upper limbs, four were located on the right side (N = 4; 57.1%) and three on the left (N = 3; 42.9%). Of the six donor bodies, the mean ± SD age at death was 75 ± 16 years. There appeared to be no association between an aberrant branching pattern and the sex or limb side of the variation. 

**Table 1:** Variations in the brachial artery by sex, body side of limb, and radial artery length above the antecubital fossa in US Midwestern donor bodies One donor body had variations in the brachial artery on both sides. Other donor bodies only had one variation. For a 1 male donor body with a left side variation, the radial artery (25.5 cm) branched from the medial side of the axillary artery and traveled laterally. All high-origin radial arteries identified in this study originated from the axillary artery.

Sex	Left or right upper limb	Radial artery length (cm)
Male	Right	19.0
Male	Left	20.0
Female	Right	21.0
Female	Left	21.6
Male	Right	21.7
Male	Left	26.5

**Figure 2:**
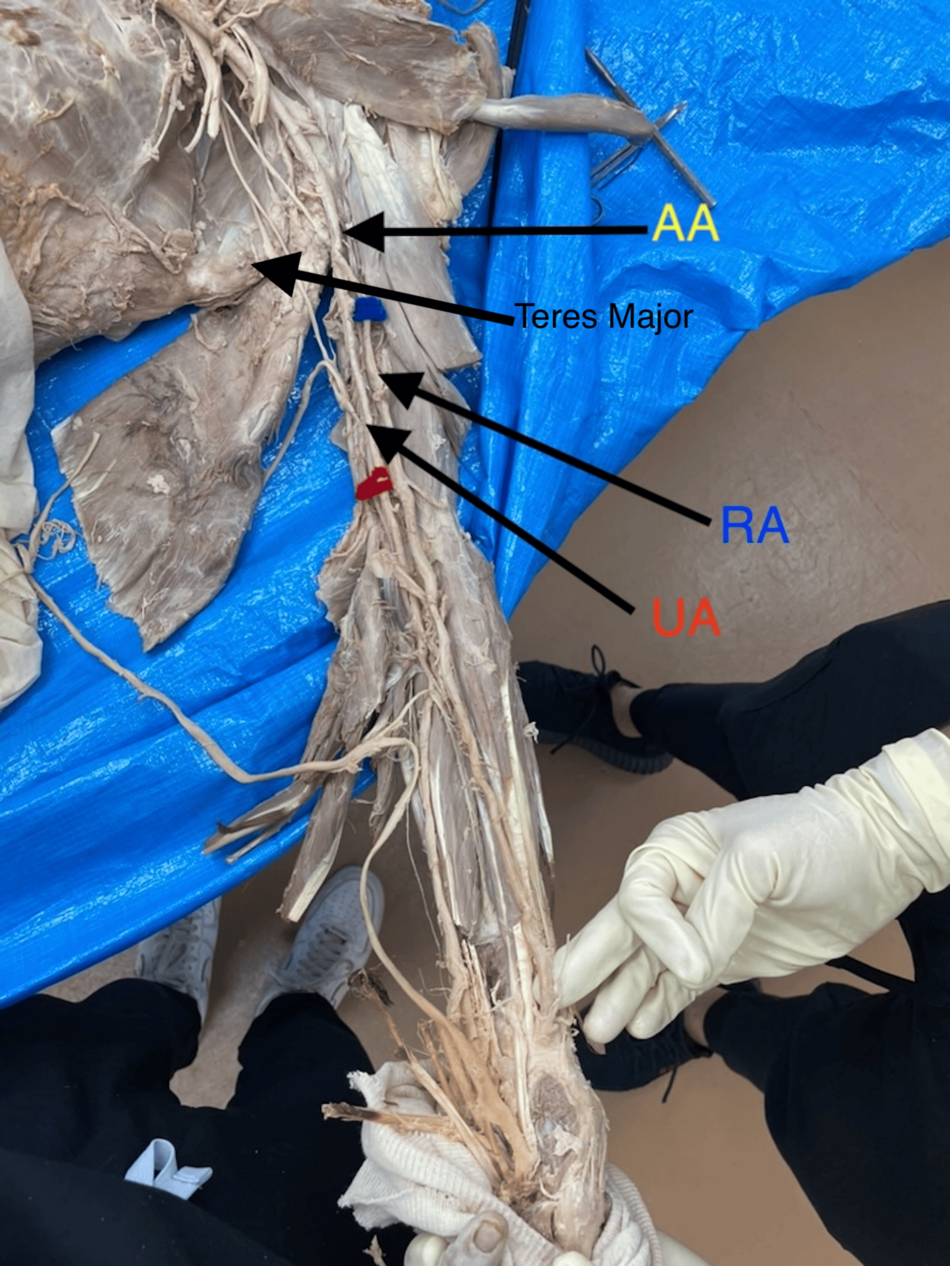
Atypical branching pattern of the brachial artery. Bifurcation of the brachial artery into radial and ulnar arteries was more proximal than the lower border of the teres major, making the axillary artery the branching point of these arteries. AA, axillary artery; RA, radial artery; UA, ulnar artery.

**Figure 3:**
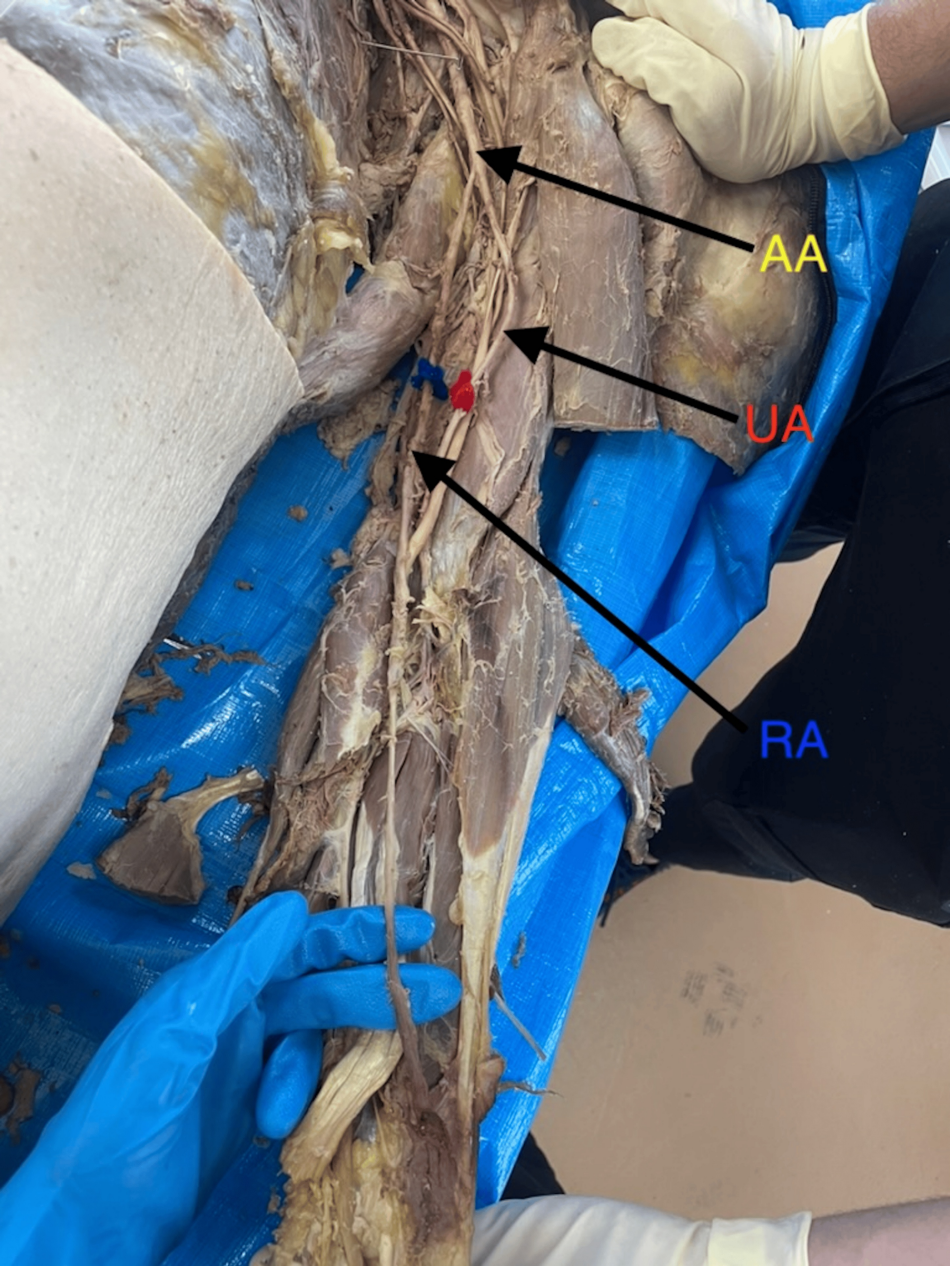
High-level bifurcation into the radial and ulnar arteries from the axillary artery. In this donor body, the radial artery branched from the medial portion of the axillary artery and then traversed the antebrachium from the medial to lateral side. AA, axillary artery; RA, radial artery; UA, ulnar artery.

All seven aberrant BAs originated proximal to the defined cubital reference point (midpoint between the medial and lateral epicondyles). The mean ± SD length of the RA from the point of bifurcation to this cubital reference point was 22.19 ± 2.58 cm, ranging from 19.0 cm to 26.5 cm (Table 1). Unlike the typical lateral origin (Figure 1), one RA originated medially from the axillary artery and crossed laterally (Figures 2, 3).

## Discussion

In the current study, we investigated variations in the BA to determine how frequently it bifurcated above the antecubital fossa. Our data indicated a variation rate of 11.5% for the seven aberrant BA in the 61 upper limbs evaluated in US Midwestern donor bodies. Our results align with prior reports, such as Glin et al. [11], which observed a 9.2% BA variation rate in a generalized adult population, and Kadel et al. [12], which found a 13.21% variation rate among Nepalese cadavers. In a study by Glin et al. [11], 120 upper extremities were evaluated, and two of the 11 aberrant BAs had an RA that originated in the axillary artery. Of the 53 upper limbs evaluated by Kadel et al. [12], 7 had aberrant BAs: one had an RA that originated from the third part of the axillary artery, three originated from the upper one-third of the arm, one originated from the middle third of the arm, and one originated from the lower part of the arm. Although previous studies [11,12] reported sex and limb side as predictors of their outcomes, no significant correlation was observed between these variables. Likewise, we did not perform any formal analyses, but there appeared to be no connection between an aberrant branching pattern and the sex or limb side of the variation. We observed no low-branching variants, unlike Kadel et al. [12], who reported such findings in a sample of 53 upper limbs. Glin et al. [11] analyzed 120 upper limbs, whereas our study examined 61. Accounting for these sample sizes helps contextualize differences in variation rates. In addition, our donor pool was composed almost entirely of White individuals, which may contribute to the observed differences when compared with the South Asian population described by Kadel et al.

Although a prospective sample size calculation was not feasible due to reliance on available donor specimens, our sample of 61 upper limbs provides comparable statistical power to previous anatomical studies reporting BA and RA variation rates (e.g., 53 limbs in Kadel et al. [12] and 120 limbs in Glin et al. [11]). Thus, our sample is sufficient to detect variation frequencies within the range reported in the literature. Given that the RA is used for a wide variety of medical procedures, the observed variation rate is clinically meaningful. It should prompt medical professionals to consider potential differences between the BA and RA. For instance, the RA is increasingly preferred for cardiac catheterization due to its ease of access and high patient comfort [13]. However, variations in the BA can affect a cardiology specialist's decision-making regarding the transradial approach, particularly in patients with a higher-branching artery. Furthermore, this variation may predispose patients to ischemia in the distal antebrachium, wrist, and hand [14]. Since the RA is one of the primary blood supplies to the lateral aspect of the distal upper limb, errors in procedures using a transradial approach may result in inadequate blood flow. Therefore, awareness of these potential variations is critical so medical professionals can take a different surgical approach in this scenario.

The lack of familiarity with branching in the BA and RA can also lead to potential radiographic diagnostic errors. Without proper knowledge of the branching pattern of the BA or the origin of the RA, diagnosing pathologies on radiographs could be challenging and potentially lead to misdiagnosis, affecting the quality of patient care. Because these variations can cause radiographic errors and misidentification of the vasculature, there may be ripple effects with invasive procedures, such as harvesting the RA for coronary artery bypass grafting [10]. Furthermore, being aware of upper limb anatomy is key to performing invasive procedures, such as retrograde catheterization, and avoiding the risk of perforating the vasculature. Retrograde catheterization is generally used during angioplasty and stent placement to resolve blockades in the coronary arteries, so a lower bifurcation of the BA into the RA and ulnar artery could cause a smaller radius of the branched vessels and a higher pressure in the blood flow, increasing the risk of perforating the RA [15].

Embryologically, the RA develops as a branch of the primitive axial artery, and variations in its definitive origin are believed to arise from persistence or regression of specific vascular channels during limb bud development. Aberrant proximal branching, such as high-origin RAs, may reflect altered remodeling pathways in the upper limb vasculature. Understanding this developmental context helps explain why these variations occur and underscores their relevance when evaluating anomalous arterial patterns in both anatomical and clinical settings.

Broader international comparisons also highlight the importance of documenting variability across different populations. Studies from European, Middle Eastern, and East Asian donor cohorts report high-origin RA frequencies ranging from approximately 5% to 18%, demonstrating considerable geographical and ethnic variation [16-20]. Our findings fall within this global spectrum and contribute region-specific data for US Midwestern donors, an underrepresented demographic in existing literature. Because anatomical variations may differ substantially across populations, localized data such as ours strengthen cumulative anatomical knowledge and inform clinical expectations in region-specific patient populations.

Clinically, identifying high-origin RAs extends beyond anatomical characterization. These variations can alter hemodynamic patterns, complicate transradial catheterization, and influence the success of graft harvesting or vascular access techniques. Misidentification on imaging may also contribute to diagnostic inaccuracies. By integrating embryological context, broader international comparisons, and procedural relevance, our findings provide meaningful interpretive insight for clinicians who increasingly rely on the RA for both diagnostic and therapeutic interventions.

Although our study data were consistent with previously reported data [11, 12], our findings are limited by the small sample size. Because our study involved a specific regional population (US Midwestern donor bodies), the lack of generalizability may also limit our findings. In addition, we did not perform formal comparisons by sex or limb side for our findings, which should also be considered a limitation.

To address these limitations, future studies should use larger sample sizes. Similarly, the study population should be expanded from a specific regional population to a more general population. Such information may yield broader, more generalizable outcomes. Furthermore, hormonal imbalances and genetic factors present during embryogenesis may contribute to aberrant vasculature origin; hence, comparisons of donor sex during data analysis may provide valuable insights into these variations. Instead of solely evaluating the proximal impact of the RA, future studies could investigate the distal RA supply to the wrist and hand to assess the effects of a distal aberrant origin.

## Conclusions

The current study evaluated variations in the bifurcation of the BA in US Midwestern donor bodies. We found an 11.5% variation rate in this branching pattern. Given the importance of variations in the BA and the origin of the RA, this finding may help inform surgeons and students about potential variations in RA and ulnar artery branching and improve healthcare delivery, particularly in interventional cardiology and radiology. Ultimately, a more thorough understanding of aberrant BA branching will support better RA access and mitigate potential complications during surgical procedures. 
